# Associations of Dietary Lipid-Soluble Micronutrients with Hepatic Steatosis among Adults in the United States

**DOI:** 10.3390/biomedicines9091093

**Published:** 2021-08-26

**Authors:** Weiwen Chai, Sarah Eaton, Heather E. Rasmussen, Meng-Hua Tao

**Affiliations:** 1 Department of Nutrition and Health Sciences, University of Nebraska—Lincoln, 1700 N 35th Street, Lincoln, NE 68583, USA; seaton5@huskers.unl.edu (S.E.); heather.rasmussen@unl.edu (H.E.R.); 2 Department of Biostatistics and Epidemiology, University of North Texas Health Science Center, 3500 Camp Bowie Boulevard, Fort Worth, TX 76107, USA

**Keywords:** α-tocopherol, retinol, vitamin D, β-carotene, carotenoids, hepatic steatosis, race/ethnicity

## Abstract

Lipid-soluble micronutrients may be beneficial to non-alcoholic fatty liver disease due to their important roles in metabolism and maintaining tissue functions. Utilizing 2017–2018 National Health and Nutrition Examination Survey, this study examined the potential overall and race/ethnicity-specific (black, Hispanic and white) associations of dietary lipid-soluble micronutrients (*α*-tocopherol, retinol, vitamin D, β-carotene and total carotenoids) with hepatic steatosis. The analysis included 4376 adults (1037 blacks, 981 Hispanics, 1549 whites) aged ≥20 years who completed the transient elastography examination with dietary data available. Odds ratios (OR) and 95% confidence intervals (95%CI) were estimated using logistic regressions. The age-adjusted prevalence of steatosis was 20.9% for blacks, 34.0% for Hispanics and 28.7% for whites. Overall, dietary α-tocopherol was inversely associated with steatosis (highest vs. lowest quartile: OR = 0.51, 95%CI = 0.35–0.74, *P_trend_* = 0.0003). The associations remained significant among blacks (highest vs. lowest tertile: OR = 0.45, 95%CI = 0.26–0.77, *P_trend_* = 0.002) and whites (highest vs. lowest tertile: OR = 0.56, 95%CI = 0.33–0.94, *P_trend_* = 0.02). Higher α-tocopherol intake was associated with lower odds of steatosis among all (*P_trend_* = 0.016) and black participants (*P_trend_* = 0.003) classified as never/rare/occasional alcohol drinkers. There was a trend suggesting higher β-carotene intake with lower odds of steatosis (*P_trend_* = 0.01). Our results suggest potential protective effects of dietary vitamin E as α-tocopherol on steatosis particularly among blacks.

## 1. Introduction

Nonalcoholic fatty liver disease (NAFLD) is becoming the most common liver disease in the United States (U.S.) [1,2,3,4], encompassing a broad spectrum of liver injury including isolated storage of fat in the liver, steatosis, and severe steatohepatitis that can progress to fibrosis, cirrhosis, liver failure or even liver cancer [2]. The etiology of NAFLD remains poorly understood and there is no accepted pharmaceutical or surgical treatment [1,2]. Therefore, modifications of diet and other lifestyle factors may play important roles in preventing the development of NAFLD as well as progression to more severe conditions once disease has developed.

Although the pathophysiology of NAFLD is complex, it has been suggested that insulin resistance and oxidative stress are likely significant contributing factors to the pathogenesis of NAFLD in all age groups [5,6,7]. Lipid-soluble micronutrients, such as carotenoids, retinol (vitamin A), vitamin D, and tocopherols (forms of vitamin E) play critical roles in metabolism and the maintenance of tissue functions [8,9,10,11]. They may also act as antioxidants neutralizing free radicals and thereby reducing oxidative stress [8,9]. Several studies have assessed the relationships between dietary factors including dietary lipid-soluble micronutrient intake and NAFLD in recent years [12,13,14,15]. For example, α-tocopherol (the predominant form of vitamin E) can limit membrane injury precipitated by reactive oxygen species and is considered as a promising antioxidant for NAFLD prevention [12]. Several observational studies have reported lower dietary or circulating α-tocopherol levels were observed in patients with NAFLD compared to normal controls [16,17]. However, randomized controlled trials showed conflicting results on the efficacy of vitamin E (as α-tocopherol) among patients with NAFLD [18,19,20]. Likewise, vitamin D deficiency is common in patients with liver diseases [21]. Epidemiological evidence shows patients with NAFLD had marked decreases in serum vitamin D levels compared to healthy individuals [22,23,24,25]. Similar to α-tocopherol, conflicting results have been reported from clinical trials examining the effects of vitamin D supplementation on the progression and severity of NAFLD [10,11]. Currently there are no solid epidemiological data on relations between dietary vitamin D intake and NAFLD. With respect to carotenoids, researchers found the odds of NAFLD was significantly reduced for the highest quartiles of intake of carotenoids (e.g., α-carotene, β-carotene, β-cryptoxanthin, and lutein/zeaxanthin) compared to the lowest. They also found serum levels of total carotenoids were inversely associated with NAFLD prevalence [13]. In addition, significantly decreased levels of circulating carotenoids such as α-carotene, β-carotene, lycopene, lutein and zeaxanthin were observed in patients with non-alcoholic steatohepatitis relative to normal controls [16].

Prior studies have suggested racial/ethnic differences in prevalence of NAFLD [3,26,27]. Hispanics, particularly experience the highest incidence and prevalence of NAFLD [3,28] and continuous annual increases in NAFLD-related mortality [29]. Although blacks had lower prevalence of NAFLD compared to whites and Hispanics [3,26], for those diagnosed with NAFLD, advanced fibrosis was nevertheless the highest for blacks among all racial/ethnic groups [3]. The associations between lipid-soluble micronutrients from diet or in circulation with health and diseases may differ among racial/ethnic groups. Our previous study using a nationally representative sample suggests there might be race/ethnicity-specific thresholds for the associations with all-cause or cause-specific mortality for serum levels of total vitamin E, *α*-tocopherol and total carotenoids [30]. To our knowledge, no studies have assessed race/ethnicity-specific relationships between lipid-soluble micronutrients and NAFLD using a nationally representative sample. Therefore, utilizing 2017–2018 National Health and Nutrition Examination Survey (NHANES), a nationally representative sample, the current study examined the potential overall and race/ethnicity-specific (black, Hispanic and white) associations of dietary intake of lipid-soluble micronutrients including *α*-tocopherol, retinol, vitamin D, β-carotene and total carotenoids with hepatic steatosis among adults in the United States. Additionally, we also investigated whether the above associations, if present varied by alcohol drinking habits.

## 2. Materials and Methods

### 2.1. Study Population

NHANES is an ongoing program of studies intended to assess the health and nutritional status of approximately 5000 adults and children in the United States each year. NHANES uses a complex, multistage, probability sampling design to select participants who are geographically dispersed and representative of the civilian, noninstitutionalized US population [31].

The 2017–2018 data were used as the liver ultrasound transient elastography were only performed in 2017–2018 NHANES examinations (besides NHANES III) to provide objective measures for two important liver disease manifestations: hepatic steatosis (fat in the liver) and fibrosis (scarring in the liver) [31]. We only used the measures for steatosis since fibrosis was not of interest in our study. The current analyses included participants aged 20 years or above. We further excluded participants who were pregnant or breastfeeding, participants whose dietary recalls were not reliable or meeting the minimum criteria, and participants with missing data on dietary intake (total energy, dietary lipid-soluble micronutrients), liver ultrasound transient elastography examination, education and body mass index (BMI). A total of 4376 participants were involved in the final analytic sample including 1037 black, 981 Hispanics and 1549 white participants.

### 2.2. Dietary Intake of Lipid-Soluble Micronutrients

The NHANES dietary interview component gathers detailed dietary intake from participants. On two separate occasions, participants reported their food and beverage intake over the past 24 h using the USDA’s Automated Multiple-Pass Method [32,33]. The two 24-h recalls were conducted in NHANES 2017 to 2018. The first dietary recall was collected in person by trained interviewers in NHANES Mobile Exam Center (MEC) and the second dietary recall was completed by trained interviewers via telephone 3–10 days after the MEC interview [32]. The data collected from each participant’s two 24-h recall interviews were coded and linked to a database of foods and beverages and their nutrient compositions. The database was used to estimate the types and amounts of food and beverages (including water) consumed, as well as to estimate energy, nutrients, and other components from those food and beverage items. For 2017–2018 NHANES cycle, dietary data were available on the following lipid-soluble micronutrients: α-tocopherol (the predominant form of vitamin E), retinol (vitamin A), vitamin D (vitamin D_2_ + vitamin D_3_), α-carotene, β-carotene, β-cryptoxanthin, lycopene, and lutein/zeaxanthin (combined measure). This paper concentrated on α-tocopherol, retinol, vitamin D, β-carotene (a major pro-vitamin A carotenoid) and total carotenoids (dietary total intake of α-carotene, β-carotene, β-cryptoxanthin, lycopene, and lutein/zeaxanthin). The current study used the first dietary recall interview because it was collected in person by trained interviewers in NHANES MEC. However, correlations between intakes of individual dietary lipid-soluble micronutrients from the first and second recall interviews were performed and they were significantly and positively correlated (*p*s < 0.0001).

### 2.3. Ascertainment of Outcomes

The elastography measurements were obtained in the NHANES MEC using FibroScan^®^ (Echosens, Cambridge, MA, USA). The device has incorporated a novel physical parameter, controlled attenuation parameter (CAP^TM^) (Echosens, Cambridge, MA, USA), which measures the ultrasound attenuation related to the presence of hepatic steatosis. All participants were asked to fast at least 3 h before the examination. The FibroScan examination procedure has been detailed in the NHANES liver Utrasound Transient Elastography Procedureds manual [34]. The above is considered as a reliable, non-invasive method and the accuracy of CAP^TM^ measurement for the detection of steatosis against biopsy has been reported in a few studies with sensitivity, specificity, and area under ROC curve being 76–79%, 71–79% and ≥80%, respectively [35,36,37]. A recent prospective study analyzed, assessed, and reported optimal CAP cutoff values to define steatosis grade/stage (S1–S3, steatosis grade/stage was initially defined by histological assessment) [38]. A threshold value of 302 dB/m was selected to define participants with steatosis (S > S1, 5% steatosis) based on the aforementioned study [38].

### 2.4. Statistical Analyses

The “Survey” procedure in SAS 9.4 software (SAS Institute, Cary, NC, USA) was used to estimate variance after incorporating the complex, multistage, clustered probability sampling design of the NHANES [39]. Characteristics and covariates were compared between those with and without steatosis using Rao–Scott chi-square test for categorical variables and Student’s *t*-test for continuous variables. The age-adjusted prevalence of steatosis was estimated, stratified by age groups using the 2000 US Census as the standard population.

Logistic regressions (proc survey logistic) were used to estimate odds ratios (OR) and 95% confidence intervals (95%CI) for associations between dietary intakes of lipid-soluble micronutrients and steatosis outcomes. For all participants (N = 4376), dietary levels of lipid-soluble micronutrients (*α*-tocopherol, retinol, vitamin D, β-carotene, total carotenoids) were categorized into quartiles using the lowest category as the reference. Two models were estimated: Model 1 was adjusted for age (continuous), sex, race/ethnicity (black, Hispanic, white, or Asian/other), education (less than high school, high school/some college, or college graduate), body mass index (BMI, continuous), smoking status (never, former, or current smoker) and daily total energy intake (continuous). Model 2 included the covariates in Model 1 and was further adjusted for alcohol drinking habits (never/rarely (never or 1 to 2 times in the last year), occasionally (3 to 11 times in the last year or once/month), sometimes (2 to 3 times/month or 1–2 times a week) or frequently (3 to 4 times a week or nearly every day or every day)), history of diabetes (yes or no) and high blood pressure (yes or no), hepatitis B virus (HBV) infection status (no infection, current/past infection, or not clear) and hepatitis C virus (HCV) infection status (no infection or current/past infection). The above analyses were repeated in three racial/ethnic groups (black, Hispanic, and white). Due to relatively small sample size for blacks, (N = 1037) and Hispanics (N = 981), dietary levels of lipid-soluble micronutrients were categorized into tertiles using the lowest tertile as the reference category. We used race/ethnicity-specific cut-points to define tertiles within the individual racial/ethnic groups. Similar to the overall analyses for all participants, we performed two models (Model 1 and Model 2) and results from the full model (Model 2) were reported. We did not perform analyses among Asians and others because of limited number of participants in these two groups. Participants were also asked about supplement use for the same 24-h period and supplement intake of vitamin D (vitamin D_2_ + vitamin D_3_), lycopene and lutein/zeaxanthin were available. For dietary vitamin D intake, we included intakes of vitamin D from both diet and supplement. Sensitivity analyses were performed after including supplement intake of lycopene and lutein/zeaxanthin into dietary consumption of total carotenoids.

The analyses for overall and race/ethnicity-specific relations stratified by participants’ alcohol drinking habits were performed by Logistic regression models (proc survey logistic). We used the question “Past 12 months how often have alcohol drink” to define participants alcohol drinking habits. We combined participants’ responses into two categories: never/rarely/occasionally vs. sometimes/frequently. A participant was classified as a never/rare/occasional alcohol drinker if the participant had 0–12 times/drinks in the past year. All statistical analyses were conducted in SAS 9.4 software (SAS Institute, Cary, NC, USA). All tests were two-sided, and *p* < 0.05 was used as the critical value for statistical significance.

## 3. Results

The age-adjusted prevalence of steatosis was 28.3% for overall population, 20.9% for blacks, 34.0% for Hispanics and 28.7% for whites. Table 1 summarizes race/ethnicity-specific characteristics by steatosis status. Among all three racial/ethnic groups (black, Hispanic and white), participants with steatosis were older and obese with a history of diabetes and high blood pressure and less likely to have a college degree compared to those without the condition (*p*s < 0.05). For Hispanics and whites, higher proportions of participants with steatosis were men and former smokers (*p*s < 0.05). Similar trends were observed among blacks for the aforementioned characteristics although the differences were not statistically significant. Blood levels of two liver enzymes, aspartate aminotransferase (AST) and alanine aminotransferase (ALT) and high-sensitive C-reactive protein (HS-CRP, indicator for systemic inflammation) were significantly higher among participants with steatosis than individuals without the disease for all racial/ethnic groups (*p*s < 0.05) except for AST among blacks. For participants with steatosis, Hispanics appeared to have the highest mean ALT and AST values. In contrast, HS-CRP levels were higher among blacks compared to other racial/ethnic groups (black: 7.7 ± 0.5 mg/L; Hispanic: 4.6 ± 0.3 mg/L; white: 5.5 ± 0.4 mg/L). With respect to dietary intake, black participants with steatosis had significantly lower intake of α-tocopherol (8.1 ± 0.4 mg/day vs. 8.9 ± 0.3 mg/day, *p* = 0.03) and total carotenoids (7385 ± 732 µg/day vs. 9261 ± 445 µg/day, *p* = 0.005) compared to their counterparts without the condition. Among Hispanics a significantly lower percentage of participants with steatosis (45.9%) reported taking any supplements relative to participants without the condition (51.7%, *p* = 0.02). Additionally, Hispanics had the lowest dietary vitamin D intake (Hispanic: 12.6 ± 0.7 µg/day vs. white: 27.7 ± 3.4 µg/day; *p* < 0.0001) followed by blacks (22.1 ± 9.0 µg/day). Dietary α-tocopherol intake was lower in blacks (8.9 ± 0.3 mg/day) than that in whites (9.6 ± 0.3 mg/day) and Hispanics (9.3 ± 0.3 mg/day) although the differences were not statistically significant.

The overall associations of dietary lipid-soluble micronutrients with steatosis for all participants (N = 4376) are shown in Table 2. Dietary α-tocopherol intake was inversely associated with steatosis in both Model 1 (*P_trent_* = 0.002) and Model 2 (fully adjusted model; *P_trent_* = 0.0003). The odds of steatosis was almost reduced to half comparing the highest to the lowest quartile of α-tocopherol intake in Model 2 (OR = 0.51, 95%CI = 0.35–0.74). For β-carotene, although there was a trend (*P_trent_* = 0.03 for Model 1, *P_trent_* = 0.01 for Model 2) suggesting higher β-carotene intake was associated with lower odds of steatosis, none of the ORs and 95%CIs were significant when comparing higher quartiles (Quartiles 2 to 4) to the lowest quartile of β-carotene intake. No significant overall associations were observed for dietary retinol, vitamin D or total carotenoids intake with steatosis (*p*s > 0.05).

Table 3 demonstrates the race/ethnicity-specific associations of dietary lipid-soluble micronutrients with steatosis. Dietary α-tocopherol intake was inversely associated with steatosis among both blacks (*P_trent_* = 0.002) and whites (*P_trent_* = 0.02) in the fully adjusted model. The odds of having steatosis was approximately 55% lower among blacks (OR = 0.45, 95%CI = 0.26–0.77) and 44% lower among whites (OR = 0.56, 95%CI = 0.33–0.94) for the highest versus the lowest tertile of α-tocopherol intake. No significant association between dietary α-tocopherol intake and steatosis was observed among Hispanics. However, the odds of having steatosis was significantly higher for the second (Tertile 2) against the lowest tertile of α-tocopherol intake (OR = 1.57, 95%CI = 1.04–2.38) among Hispanics. No significant associations of dietary retinol, vitamin D, β-carotene or total carotenoids with steatosis were observed among the individual racial/ethnic groups although the odds of steatosis were significantly higher for the second against the lowest tertile for β-carotene (OR = 1.60, 95%CI = 1.08–2.39) among whites and for total carotenoids (OR = 1.49, 95%CI = 1.06-2.09) among blacks. Results did not change materially after including supplement intake of lycopene and lutein/zeaxanthin (the two carotenoids in the current study that had supplement intake information) into the intake of total carotenoids. Further adjusting for dietary supplement intake (yes or no) did not significantly change the results.

The overall and race/ethnicity-specific associations between dietary micronutrients levels and steatosis stratified by alcohol drinking habits (never/rarely/occasionally vs. sometimes/frequently) are shown in Table 4. For the overall population, higher α-tocopherol intake was associated with reduced odds of steatosis among participants who were characterized as never/rare/occasional drinkers (the highest quartile vs. the lowest quartile: OR = 0.49, 95%CI = 0.27–0.89; *P_trent_* = 0.016). A similar but weaker trend between α-tocopherol intake and steatosis was found among participants who were sometimes/frequent alcohol drinkers (*P_trend_* = 0.06). Also, there was a trend (*P_trent_* = 0.007) suggesting an inverse association between β-carotene intake and odds of steatosis among never/rare/occasional drinkers although odds and 95%CIs for the higher quartiles against the lowest were not statistically significant. In terms of race/ethnicity-specific associations, dietary α-tocopherol intake was inversely associated with steatosis (*P_trend_* = 0.003) among blacks who never, rarely or occasionally drank alcohol. There was approximately 63% reduction in odds of having steatosis for the highest versus the lowest tertile of α-tocopherol intake (OR = 0.37; 95%CI = 0.18–0.73). Although not statistically significant, a similar trend showing increased α-tocopherol intake with lower odds of steatosis was observed among white participants who were sometimes/frequent alcohol drinkers (*P_trend_* = 0.09). In addition, among whites who were never/rare/occasional drinkers, there appeared to be an inverse trend between β-carotene intake and odds of steatosis (*P_trend_* = 0.01) but the odds of the disease was higher for the second versus the lowest tertile of β-carotene intake (OR = 1.81, 95%CI = 1.04–3.17). For Hispanics classified as sometimes/frequent drinkers, the odds of steatosis was higher for the second tertile against the lowest of α-tocopherol intake (OR = 2.62, 95%CI = 1.17–5.86). No significant associations of dietary intakes of retinol, vitamin D or total carotenoids with steatosis were observed after stratification for alcohol drinking habits except for a borderline, non-statistically significant trend suggesting higher vitamin D intake with lower odds of steatosis among Hispanics classified as sometimes/frequent alcohol drinkers (*P_trend_* = 0.08).

## 4. Discussion

Utilizing data from the recent NHANES cycle that had transient elastography measures available for objectively detecting hepatic steatosis, the current results suggest that higher intake of dietary α-tocopherol was associated with lower odds of steatosis. The protective association of dietary α-tocopherol with steatosis remained significant among blacks and whites. When stratified by alcohol drinking habits, we also found dietary intake of α-tocopherol was inversely associated with the disease among those classified as never/rare/occasional drinkers for the overall study population as well as among blacks who belonged to the “never/rare/occasional” category.

Oxidative stress is considered as one of the key mechanisms of hepatocellular damage and pathogenesis of NAFLD [5,6,7,40,41]. Vitamin E, particularly α-tocopherol due to its anti-oxidative properties has been well studied as a treatment for hepatic steatosis. Two well-known randomized clinical trials, “Pioglitazone, Vitamin E, or Placebo for Nonalcoholic Steatohepatitis’ (PIVENS) trial [42], and the “Treatment of NAFLD in Children” (TONIC) trial [43] both demonstrated significant improvements in liver histology with vitamin E treatment compared to placebo. In addition, a recent systematic review with meta-analysis of randomized controlled trials with vitamin E treatment concluded that adjuvant vitamin E therapy provides significant biochemical and histological improvements in adult patients with NAFLD although the short trail durations limit the conclusion on the safety and efficacy of proposed treatments [19]. Data from observational studies are scarce. Several observational studies showed protective associations of dietary vitamin E intake with NAFLD [44,45,46,47]. However, these studies had limited sample size and/or less diverse study populations regarding race/ethnicity. Therefore, we assessed overall and race/ethnicity-specific associations between dietary α-tocopherol intake and hepatic steatosis using a representative sample of the US population with a large sample size. Our results in the overall study population and in blacks and whites were in agreement with previous randomized trials [19,42,43] and observational studies [44,45,46,47] showing the potential protective effects of vitamin E (as α-tocopherol) in relation to NAFLD. In particular, the protective association was more prominent in blacks. Meanwhile, Hispanics had the highest prevalence of NAFLD [3,28] but we did not observe a significant association between α-tocopherol and steatosis in this racial/ethnic group. In fact, the second tertile of α-tocopherol intake had significantly higher odds of steatosis compared to the lowest tertile among Hispanics, which warrants a further investigation in future studies. Furthermore, research has suggested genetic variations may in part contribute to the racial/ethnic differences in the prevalence of NAFLD [48,49,50,51,52]. For example, the *PNPLA3* “G” risk allele was found more common in Hispanics than other racial/ethnic groups [49,50,51,52], and thereby may have an impact on NAFLD risk in Hispanics [49,53]. Thus, it would be interesting for future studies to investigate whether genetic variations may also play a role in racial/ethnic differences in repose to the potential beneficial effects of α-tocopherol on steatosis as observed in the current study.

Increasing evidence suggests the importance of vitamin D as physiological regulator beyond its classical role in bone and calcium homeostasis [54]. Results from observational studies show patients with NAFLD had lower levels of serum 25-hydroxy-vitamin D (25(OH)D, indicator for vitamin D status in the human body) compared to individuals without NAFLD [22,23,24,25] including one recent study utilizing NHANES III (1988–1994) that reported serum 25(OH)D levels were independently and inversely associated with severity of NAFLD [25]. In our study, we did not observe significant associations between dietary vitamin D intake (vitamin D from diet and supplement) and steatosis among all participants as well as participants stratified by race/ethnicity. In human body the majority (90%) of vitamin D is derived from cutaneous conversion of 7-dehydrocholesterol to vitamin D_3_ through exposure to sunlight and only about 10% is from dietary intake [54]. This may in part explain the discrepancies in the results between serum and dietary vitamin D in relation to NAFLD, that is, NAFLD was associated with lower serum vitamin D concentrations as suggested by previous studies but not related to dietary vitamin D intake observed in our study. That said, consuming an adequate amount of vitamin D from diet and/or supplement is nevertheless relevant for high-risk populations, for example blacks and Hispanics among whom high prevalence of vitamin D deficiency has been reported [55]. In fact, we observed Hispanics had the lowest vitamin D intake (from diet and supplement) (12.6 ± 0.7 µg/day) among the racial/ethnic groups in the current study; the value was more than two-fold lower compared to whites (27.2 ± 3.3 µg/day).

Healthful eating patterns, including high fruit and vegetable consumption, have been associated with decreased risk for metabolic diseases and overall improved health [56]. Because of this, it is hypothesized that healthful dietary intake could also have protective effects on the development and progression of NAFLD. Carotenoids are naturally occurring pigments found in various fruits and vegetables, plants, algae and bacteria [57]. Thus, higher levels of dietary carotenoids may suggest that a person has consumed an adequate amount of fruits and vegetables [8,9]. Limited studies have assessed the potential protective effects of various carotenoids on NAFLD. Using NHANES, Christensen et al. found levels of dietary and serum carotenoids such as α-carotene, β-carotene, β-cryptoxanthin, and lutein/zeaxanthin were inversely associated with the odds of NAFLD [13]. Among carotenoids, β-carotene is the most abundant carotenoid in the liver and has the highest pro-vitamin A activity [57,58]. Experimental research has demonstrated the protective effects of β-carotene on NAFLD in rats [59]. In humans, studies found lower serum β-carotene levels or β-carotene to retinol ratio were associated with severity of NAFLD [60,61] while high β-carotene concentrations were associated with improvement of the condition [62]. In our study we did not observe significant associations overall or within individual racial/ethnic groups for dietary total carotenoids. However, we observed a trend with higher β-carotene intake and lower odds of steatosis for all participants without stratifications and participants who were classified as never, rare or occasional alcohol drinkers. Similar trend was also observed for white participants who belonged to the “never/rare/occasional drinkers” category although there appeared a threshold effect with odds of steatosis being higher for the second versus the lowest tertile of β-carotene intake. No associations were found between dietary retinol (vitamin A) intake and steatosis. Although future research is necessary to fully investigate the potential protective effects of β-carotene on steatosis and the optimum range of intake of this nutrient needs to be established since high dosage of β-carotene intake was linked to increased incidence of cancer and mortality [63,64], the current results and findings from previous studies confirm the recommendation for promoting dietary consumption of antioxidants such as β-carotene and other carotenoids through fruits and vegetables.

In our study, we observed dietary α-tocopherol intake was inversely associated with odds of steatosis among those who classified as never, rare or occasional alcohol drinkers for the overall study population as well as for black participants who never, rarely or occasionally drank alcohol. However, a similar but weaker pattern of association was also observed among those who drank alcohol more frequently (sometimes/frequent drinkers) although the results did not reach statistical significance possibly due to the smaller number of participants in this category (sometimes/frequent drinkers: N = 1594 vs. never/rare/occasional drinkers: N = 2668). Also, we did not observe interactions between alcohol drinking habits and α-tocopherol intake in relation to steatosis (*p* > 0.05). In addition, we found a trend suggesting higher dietary β-carotene intake with lower odds of steatosis among never, rare or occasional alcohol drinkers for the overall population and whites. The NHANES self-report alcohol behavior questionnaire assessed the frequency of alcohol drinking. There was no information regarding the exact amount of alcohol an individual consumed each time, which may cause misclassification when categorizing participants solely based upon how frequently one drank alcohol beverages. Therefore, the above results need to be confirmed in future studies with detailed information on individuals’ alcohol consumption.

One thing that needs to be noticed from our study is that the protective associations between dietary α-tocopherol intake and steatosis were consistent among black participants, suggesting the effects of vitamin E (as α-tocopherol) on NAFLD maybe race/ethnicity-specific with more promising results observed in blacks. The possible explanation could be blacks maybe more susceptible to α-tocopherol treatment. Using NHANES 1999 to 2000, a pervious study reported African-Americans had the lowest serum α-tocopherol levels among all the racial/ethnic groups (African-Americans, Mexican-Americans and whites) examined [65]. We found dietary α-tocopherol intake was also lower in blacks (8.9 ± 0.3 mg/day) than that in whites (9.6 ± 0.3 mg/day) and Hispanics (9.3 ± 0.3 mg/day). Conversely, the potential beneficial effect of α-tocopherol on steatosis was nevertheless found in blacks. Interestingly, we also observed blacks with steatosis had higher HS-CRP (indicator for systemic inflammation) levels (7.7 ± 0.5 mg/L) compared to their counterparts in other racial/ethnic groups (Hispanics: 4.6 ± 0.3 mg/L; whites: 5.5 ± 0.4 mg/L). Inflammation has been linked to NAFLD [66,67] and the progression to more severe liver conditions such as cirrhosis and caner [68]. Thus, it would be interesting and meaningful for future studies to elucidate the interrelationships and mechanisms between α-tocopherol, inflammation and steatosis among blacks. The 2017–2018 NHANES cycle does not have information on supplement vitamin E intake. Therefore, it is uncertain whether blacks also had lower dietary vitamin E supplementation compared to other racial/ethnic groups. However, one study using NHANES 2009–2012 revealed differential use of dietary supplements across racial/ethnic groups with blacks (40%) and Hispanics (36%) having lower prevalence of supplement use compared to whites (61%) [69]. In the current study using NHANES 2017–2018, we also observed a lower percentage of blacks (47.4%) who reported taking supplements (any kind) relative to whites (61.9%). Further studies focusing on blacks are needed to confirm our results and identify optimum intake range of vitamin E for this racial/ethnic group. In addition, detailed dietary information on vitamin E consumption including intake of α-tocopherol from both diet and supplement and different forms (e.g., α-tocopherol vs. γ-tocopherol) and sources of tocopherols (e.g., natural products vs. synthetic compounds) should be collected when assessing relationships between vitamin E and steatosis.

To our knowledge, the current study was the first to investigate both overall and race/ethnicity-specific relations between dietary intakes of lipid-soluble micronutrients and hepatic steatosis using a representative sample of the U.S. population. The strengths of this study included using NHANES data with nationally representative samples and a relatively large number of adults with ultrasound transient elastography examination, providing the power to detect weaker associations. Additionally, our study utilized the liver ultrasound transient elastography which is considered as a more objective measure for hepatic steatosis [31]. Our study had several limitations. First, due to the nature of cross-sectional studies, the temporal sequences may not be clear. Second, although the transient elastography measurement is a widely used non-invasive method to assess liver steatosis [35,36,37], it can be limited by fatness of a patient, the presence of perihepatic ascites, and limited selection of an appropriate sampling area [70]. Third, misclassification may have occurred in the analyses since there is no well-defined cutoff for steatosis utilizing the transient elastography measurement. However, the cutoff (302 dB/m) used in our study was recommended by Eddowes et al. who established this value using liver biopsy, which is considered the gold standard for NAFLD diagnosis [38]. The 24-h dietary recall used in NHANES has been extensively evaluated [32]; however, self-reported dietary recall is likely to have both random and systematic errors [71], and a one-time, 24-h dietary recall may not capture long-term dietary exposures. Further, our analyses did not include 24-h dietary supplement intake (except for vitamin D) as there were no available data on supplements such as α-tocopherol, retinol and most of the carotenoids for that day. However, further adjustment for participants’ overall supplement intake status (taking supplement: yeas or no) did not change the results substantially. Lastly, due to the unavailability of data on circulating lipid-soluble micronutrients in the 2017–2018 NHANES cycle, we did not further assess the associations of serum concentrations of lipid-soluble micronutrients with steatosis, which would provide a more complete picture for understanding the relationships between micronutrients and NAFLD.

## 5. Conclusions

In the current study, dietary intake of α-tocopherol was inversely associated with odds of hepatic steatosis. The inverse association remained significant among blacks and whites. After stratification by individuals’ alcohol drinking habits, higher dietary α-tocopherol intake was associated with lower odds of steatosis among those who were classified as never, rare, or occasional alcohol drinkers for the overall study population as well as for black participants. In addition, there was a trend suggesting higher β-carotene intake with lower odds of steatosis. No significant associations were observed for other dietary lipid-soluble micronutrients (retinol, vitamin D, total carotenoids) in relation to steatosis. Our results suggest a potential protective effect of dietary vitamin E as α-tocopherol on hepatic steatosis particularly among blacks. Due to the cross-sectional natural of the study design, our results especially the unique findings between dietary α-tocopherol intake and steatosis among blacks should be confirmed by prospective cohorts and randomized trials in the future.

## Figures and Tables

**Table 1:** Characteristics of Study Participants by Hepatic Steatosis Status and Race/Ethnicity.

Characteristics	Black				Hispanic				White			
	All	Non-Steatosis	Steatosis	*p* ^a^	All	Non-Steatosis	Steatosis	*p* ^a^	All	Non-Steatosis	Steatosis	*p* ^a^
N	1037	804	233		981	626	355		1549	1055	494	
Age (y)	45.8 ± 0.6	44.6 ± 0.7	50.4 ± 1.1	0.0001	43.0 ± 0.9	41.7 ± 1.0	45.7 ± 1.1	0.0002	50.3± 0.9	49.0± 1.0	53.6± 0.9	0.0001
Sex, (%)				0.39				0.001				<0.0001
Men	47.4	46.2	51.7		51.0	46.6	59.4		49.6	44.6	62.0	
Women	52.6	53.8	48.3		50.0	53.4	40.6		50.4	55.4	38.0	
Education (%)				0.04				0.01				0.0003
Below high school	11.5	11.1	12.9		26.8	28.1	24.3		6.1	6.0	6.6	
High school/some college	67.4	66.1	72.2		57.3	54.3	63.2		59.0	56.5	65.3	
College graduate	21.1	22.8	14.9		15.9	17.7	12.5		34.9	37.5	28.1	
Body mass index (kg/m^2^)	31.1 ± 0.3	29.3 ± 0.2	38.1 ± 0.6	<0.0001	30.4 ± 0.3	28.4 ± 0.4	34.3 ± 0.6	<0.0001	29.6 ± 0.4	29.0 ± 0.3	35.2 ± 0.6	<0.0001
Obese (≥30 kg/m^2^) (%)	49.1	38.6	88.7	<0.0001	45.7	32.1	72.0	<0.0001	42.5	29.0	75.9	<0.0001
Smoke status (%)				0.08				0.03				<0.0001
Never	62.8	64.6	55.8		64.9	67.5	59.9		53.9	56.6	47.2	
Former	16.0	14.5	21.7		22.4	19.4	28.2		28.6	25.6	36.2	
Current	21.2	20.9	22.5		12.7	13.1	11.9		17.5	17.8	16.6	
Alcohol drinking habits in past 12 months (%)				0.42				0.33				0.07
Never/rarely	34.8	33.7	38.4		33.8	34.3	32.8		30.6	28.1	37.0	
Occasionally	24.4	24.2	25.4		22.6	23.1	21.4		21.7	21.2	22.8	
Sometimes	27.0	27.8	24.1		34.9	35.3	34.1		31.8	33.7	27.0	
Frequently	13.8	14.3	12.1		8.7	7.3	11.5		15.9	17.0	13.2	
Diabetes (%)	14.8	11.1	28.8	<0.0001	12.0	7.5	20.8	<0.0001	13.4	7.2	29.0	<0.0001
High blood pressure (%)	40.4	35.2	59.4	<0.0001	22.1	17.3	31.5	<0.0001	33.1	25.7	51.8	<0.0001
HBV infection (%) ^b^	6.5	6.1	8.0	0.69	2.8	2.1	4.2	0.18	2.7	2.1	3.9	0.11
HCV infection (%) ^c^	2.8	2.7	3.2	0.34	1.7	1.3	2.4	0.12	3.2	3.5	2.3	0.50
AST (IU/L) ^d^	21.5 ± 0.4	21.1 ± 0.6	23.0 ± 0.9	0.08	22.6 ± 0.6	20.9 ± 0.4	26.0 ± 1.2	<0.0001	22.4 ± 0.5	21.7 ± 0.6	24.2 ± 1.0	<0.0001
ALT (IU/L) ^e^	20.1 ± 0.6	18.6 ± 0.7	25.6 ± 0.9	<0.0001	25.9 ± 0.7	22.3 ± 0.7	32.8 ± 1.4	<0.0001	23.2 ± 0.7	20.7 ± 0.6	29.5 ± 1.6	<0.0001
HS-CRP (mg/L) ^f^	4.6 ± 0.1	3.8 ± 0.2	7.7 ± 0.5	<0.0001	3.8 ± 0.3	3.4 ± 0.3	4.6 ± 0.3	0.002	3.7 ± 0.2	3.0 ± 0.3	5.5 ± 0.4	<0.0001
Dietary intake												
Total energy (kcal)	2126 ± 33	2127 ± 40	2121 ± 86	0.84	2233 ± 31	2186 ± 39	2324± 65	0.40	2209 ± 27	2172 ± 38	2302 ± 47	0.53
α-Tocopherol (mg/day)	8.9 ± 0.3	8.9 ± 0.3	8.1 ± 0.4	0.03	9.3 ± 0.3	9.4 ± 0.4	9.1 ± 0.3	0.34	9.6 ± 0.3	9.8 ± 0.3	8.9 ± 0.3	0.16
Retinol (µg/day)	343 ± 17	345 ± 21	336 ± 26	0.42	379 ± 19	375 ± 24	387 ± 26	0.71	444 ± 9	448 ± 11	436 ± 25	0.64
Vitamin D (µg/day) ^g^	22.1 ± 7.0	22.0 ± 8.6	22.6 ± 7.3	0.45	12.6 ± 0.7	13.1 ± 0.7	11.5 ± 1.4	0.61	27.2 ± 3.4	27.0 ± 4.7	27.7 ± 3.4	0.94
β-Carotene (µg/day)	2328 ± 242	2463 ± 282	1821 ± 230	0.48	2508 ± 235	2659 ± 269	2215 ± 308	0.24	2429 ± 200	2452 ± 217	2371 ± 340	0.53
Tot carotenoid (µg/day) ^h^	8868 ± 413	9261 ± 445	7385 ± 732	0.005	10743 ± 745	11036 ± 942	10175 ± 888	0.68	9181 ± 403	9344 ± 509	8774 ± 724	0.42
Take supplement (%)	47.4	46.0	52.5	0.28	49.7	51.7	45.9	0.02	61.1	61.7	59.6	0.47

Note: Values are presented as weighted mean ± SE and weighted percentage (%). ^a^ *p* values for differences between participants with steatosis and participants without steatosis within the individual racial/ethnic groups using t tests for continuous variables and chi-squared test for categorical variables; ^b^ HBV = hepatitis B virus; ^c^ HCV = hepatitis C virus; ^d^ AST = Aspartate aminotransferase; ^e^ ALT = Alanine aminotransferase; ^f^ HS-CRP = high-sensitive C-reactive protein; ^g^ Daily vitamin D intake includes vitamin D intake from diet and supplement; ^h^ Tot carotenoids = total carotenoids.

**Table 2:** Overall Odds Ratio and 95% Confidence Interval for Hepatic Steatosis by Quartiles (Q1-Q4) of Dietary Micronutrients.

Micronutrients	OR (95% CI) ^a^				*P_trend_* ^a^
	Q1	Q2	Q3	Q4	
α-Tocopherol (mg/day)					
Quartile range	<4.4	4.4–6.9	6.9–10.5	≥10.5	
Number of participants	964	1031	1113	1268	
Number of cases	293	299	344	361	
Model 1 ^b^	1.00	0.76 (0.58–0.99)	0.81 (0.54–1.21)	0.54 (0.39–0.76)	0.002
Model 2 ^c^	1.00	0.81 (0.60–1.10)	0.83 (0.55–1.26)	0.51 (0.35–0.74)	0.0003
Retinol (µg/day)					
Quartile range	<161.0	161.0–333.0	333.0–561.0	≥561.0	
Number of participants	1268	1118	1027	963	
Number of cases	360	335	307	295	
Model 1 ^b^	1.00	1.04 (0.75–1.44)	0.74 (0.50–1.11)	0.88 (0.54–1.46)	0.53
Model 2 ^c^	1.00	1.00 (0.71–1.42)	0.73 (0.48–1.09)	0.86 (0.52–1.44)	0.51
Vitamin D (µg/day) ^d^					
Quartile range	<1.7	1.7–4.8	4.8–13.3	≥13.3	
Number of participants	1179	1053	809	1335	
Number of cases	320	304	258	415	
Model 1 ^b^	1.00	0.96 (0.71–1.31)	1.00 (0.70–1.45)	1.09 (0.78–1.54)	0.41
Model 2 ^c^	1.00	0.93 (0.67–1.30)	0.98 (0.67–1.45)	0.99 (0.69–1.43)	0.87
β-Carotene (µg/day)					
Quartile range	<217.0	217.0–615.0	615.0–1924.5	≥1924.5	
Number of participants	857	995	1202	1322	
Number of cases	243	326	378	350	
Model 1 ^b^	1.00	1.08 (0.81–1.45)	1.14 (0.86–1.53)	0.85 (0.63–1.15)	0.03
Model 2 ^c^	1.00	1.16 (0.87–1.56)	1.20 (0.86–1.66)	0.87 (0.65–1.16)	0.01
Total Carotenoids (µg/day)					
Quartile range	<1429.5	1429.5–4380.5	4380.5–10563.0	≥10563.0	
Number of participants	935	1039	1138	1264	
Number of cases	289	296	344	368	
Model 1 ^b^	1.00	0.92 (0.64–1.32)	0.87 (0.56–1.36)	0.96 (0.66–1.39)	0.93
Model 2 ^c^	1.00	1.00 (0.69–1.45)	0.86 (0.52–1.43)	0.99 (0.69–1.43)	0.91

^a^ Odds ratio (OR), 95 confidence interval (95% CI) and *P_trend_* values were estimated using logistic regression (Proc Survey Logistic); ^b^ Model 1 was adjusted for age, race/ethnicity, sex, body mass index, education, smoking status and total energy intake; ^c^ Model 2 was adjusted for covariates in Model 1 and was additionally adjusted for alcohol drink habits, history of diabetes, history of hypertension, Hepatitis B infection status and Hepatitis C infection status; ^d^ Daily vitamin D intake includes vitamin D intake from diet and supplement.

**Table 3:** Race/Ethnicity-Specific Odds Ratio and 95% Confidence Interval for Hepatic Steatosis by Tertles (T1-T3) of Dietary Micronutrients.

Micronutrients		Black			Hispanic			White	
	T1	T2	T3	T1	T2	T3	T1	T2	T3
α-Tocopherol (mg/day)									
Tertile range	<5.3	5.3–9.5	≥9.5	<5.6	5.6–10.3	≥10.3	<6.1	6.1–10.2	≥10.2
Participants (N)	347	331	359	343	338	300	574	481	494
Cases (N)	83	78	72	123	124	108	182	157	155
OR (95% CI) ^a^	1.00	0.96 (0.56–1.64)	0.45 (0.26–0.77)	1.00	1.57 (1.04–2.38)	1.23 (0.81–1.88)	1.00	0.99 (0.67–1.47)	0.56 (0.33–0.94)
	*P_trend_*^a^ = 0.002			*P_trend_*^a^ = 0.59			*P_trend_*^a^ = 0.02		
Retinol (µg/day)									
Tertile range	<135.2	135.2–364.2	≥364.2	<182.5	182.5–427.8	≥427.8	<246.2	246.2–491.2	≥491.2
Participants (N)	341	338	358	319	344	318	505	482	562
Cases (N)	67	91	75	117	119	119	158	156	180
OR (95% CI) ^a^	1.00	1.20 (0.83–1.73)	0.94 (0.48–1.84)	1.00	0.88 (0.60–1.34)	1.20 (0.66–2.22)	1.00	0.73 (0.42–1.27)	0.77 (0.40–1.50)
	*P_trend_*^a^ = 0.75			*P_trend_*^a^ = 0.47			*P_trend_*^a^ = 0.52		
Vitamin D (µg/day) ^b^									
Tertile range	<1.5	1.5–6.7	≥6.7	<1.9	1.9–6.2	≥6.2	<2.9	2.9–15.9	≥15.9
Participants (N)	319	344	374	301	317	363	489	517	543
Cases (N)	57	83	93	107	122	126	154	170	170
OR (95% CI) ^a^	1.00	1.03 (0.72–1.47)	0.99 (0.53–1.83)	1.00	1.03 (0.57–1.86)	0.86 (0.55–1.36)	1.00	0.96 (0.64–1.42)	0.91 (0.57–1.45)
	*P_trend_*^a^ = 0.93			*P_trend_*^a^ = 0.37			*P_trend_*^a^ = 0.68		
β-Carotene (µg/day)									
Tertile range	<333.1	333.1–1254.2	≥1254.2	<554.8	554.8–1836.6	≥1836.6	<411.1	411.1–1600.6	≥1600.6
Participants (N)	348	328	361	313	332	336	567	499	483
Cases (N)	69	89	75	116	124	115	184	170	140
OR (95% CI) ^a^	1.00	1.39 (0.94–2.07)	1.07 (0.59–1.95)	1.00	0.88 (0.54–1.45)	0.74 (0.51–1.07)	1.00	1.60 (1.08–2.39)	0.99 (0.75–1.31)
	*P_trend_*^a^ = 0.77			*P_trend_*^a^ = 0.11			*P_trend_*^a^ = 0.22		
Total carotenoids (µg/day)									
Tertile range	<2009.8	2009.8–7732.4	≥7732.4	<3464.6	3464.6–10075.0	≥10075.0	3005.4	3005.4–9489.2	≥9489.2
Participants (N)	352	338	347	340	329	312	562	508	479
Cases (N)	72	90	71	129	114	112	183	157	154
OR (95% CI) ^a^	1.00	1.49 (1.06–2.09)	1.08 (0.60–1.93)	1.00	0.84 (0.57–1.23)	0.90 (0.48–1.69)	1.00	0.96 (0.52–1.77)	1.17 (0.67–2.03)
	*P_trend_*^a^ = 0.77			*P_trend_*^a^ = 0.83			*P_trend_*^a^ = 0.53		

^a^ Odds ratio (OR), 95% confidence interval (95% CI) and *P_trend_* values were estimated by logistic regression (Proc Survey Logistic). Analyses were adjusted for age, sex, education, body mass index, smoking status, total energy intake, alcohol drinking habits, hepatitis B virus infection, hepatitis C virus infection, history of diabetes and history of high blood pressure; ^b^ Daily vitamin D intake includes vitamin D intake from diet and supplement.

**Table 4:** Overall and Race/Ethnicity-Specific Associations of Dietary Micronutrients with Hepatic Steatosis by Alcohol Drinking Habits.

Race/Ethnicity		Never/Rare/Occasional Alcohol Drinker				Sometimes/Frequent Alcohol Drinker			
	Quartile (Q1–Q4)/Tertile (T1–T3)	Participants (N)	Cases (N)	OR (95%CI) ^a^	*P_trend_* ^a^	Participants (N)	Cases (N)	OR (95%CI) ^a^	*P_trend_* ^a^
All participants	α-Tocopherol (mg/day)								
	Q1 (<4.4)	646	201	1.00		294	83	1.00	
	Q2 (4.4–6.9)	646	197	0.83 (0.55–1.26)		361	99	0.98 (0.67–1.42)	
	Q3 (6.9–10.5)	655	209	0.78 (0.46–1.33)		430	131	1.02 (0.54–1.94)	
	Q4 (≥10.5)	721	220	0.49 (0.27–0.89)	0.016	509	131	0.60 (0.32–1.13)	0.06
	Retinol (µg/day)								
	Q1 (<161.0)	777	229	1.00		449	117	1.00	
	Q2 (161.0–333.0)	681	214	1.08 (0.72–1.60)		412	117	1.13 (0.70–1.83)	
	Q3 (333.0–561.0)	613	191	0.92 (0.48–1.74)		385	112	0.63 (0.41–0.97)	
	Q4 (≥561.0)	597	193	1.02 (0.59–1.77)	0.95	348	98	0.76 (0.34–1.69)	0.38
	Vitamin D (µg/day) ^b^								
	Q1 (<1.7)	707	208	1.00		440	105	1.00	
	Q2 (1.7–4.8)	619	189	1.22 (0.79–1.90)		407	108	0.70 (0.37–1.31)	
	Q3 (4.8–13.3)	478	150	0.89 (0.54–1.44)		305	102	1.11 (0.65–1.91)	
	Q4 (≥13.3)	864	280	0.92 (0.57–1.50)	0.47	442	129	1.15 (0.63–2.11)	0.28
	β-Carotene (µg/day)								
	Q1 (<217.0)	560	165	1.00		275	72	1.00	
	Q2 (217.0–615.0)	597	204	1.08 (0.72–1.61)		375	116	1.54 (0.87–2.71)	
	Q3 (615.0–1924.5)	722	236	1.36 (0.89–2.08)		447	136	1.19 (0.76–1.86)	
	Q4 (≥1924.5)	789	222	0.78 (0.54–1.14)	0.007	497	120	1.13 (0.62–2.08)	0.47
	Total carotenoids (µg/day)								
	Q1 (<1429.5)	609	200	1.00		303	81	1.00	
	Q2 (1429.5–4380.5)	630	179	0.92 (0.55–1.54)		381	115	1.49 (1.06–2.10)	
	Q3 (4380.5–10563.0)	689	218	0.93 (0.50–1.74)		415	114	0.95 (0.42–2.18)	
	Q4 (≥10563.0)	740	230	0.91 (0.67–1.23)	0.66	495	134	1.36 (0.67–2.79)	0.62
Black participants	α-Tocopherol (mg/day)								
	T1 (<5.3)	211	54	1.00		126	29	1.00	
	T2 (5.3–9.5)	193	46	0.94 (0.52–1.71)		129	31	0.92 (0.37–2.30)	
	T3 (≥9.5)	206	45	0.37 (0.18–0.73)	0.003	141	24	0.61 (0.24–1.56)	0.29
	Retinol (µg/day)								
	T1 (<135.2)	198	44	1.00		132	22	1.00	
	T2 (135.2–364.2)	185	47	1.15 (0.78–1.69)		145	43	1.10 (0.55–2.19)	
	T3 (≥364.2)	227	54	1.17 (0.68–2.01)	0.65	119	19	0.41 (0.11–1.44)	0.13
	Vitamin D (µg/day) ^b^								
	T1 (<1.5)	182	38	1.00		129	19	1.00	
	T2 (1.5–6.7)	195	47	0.74 (0.48–1.14)		137	32	1.25 (0.79–1.98)	
	T3 (≥6.7)	233	60	0.94 (0.49–1.83)	0.75	130	33	0.88 (0.30–2.59)	0.68
	β-Carotene (µg/day)								
	T1 (<333.1)	208	46	1.00		129	23	1.00	
	T2 (333.1–1254.2)	183	51	1.02 (0.60–1.74)		138	37	2.07 (0.79–5.40)	
	T3 (≥554.8)	219	48	0.87 (0.41–1.87)	0.65	129	24	1.46 (0.72–2.99)	0.94
	Total carotenoids (µg/day)								
	T1 (<2009.8)	211	48	1.00		133	24	1.00	
	T2 (2009.8–7732.4)	189	52	1.47 (0.74–2.95)		141	38	1.74 (0.91–3.33)	
	T3 (≥7732.4)	210	45	0.80 (0.39–1.64)	0.22	122	22	2.07 (0.92–4.66)	0.13
Hispanic participants	α-Tocopherol (mg/day)								
	T1 (<5.6)	226	76	1.00		106	40	1.00	
	T2 (5.6–10.3)	200	65	1.18 (0.66–2.14)		130	57	2.62 (1.17–5.86)	
	T3 (≥10.3)	171	64	1.20 (0.77–1.87)	0.45	117	40	1.57 (0.85–2.89)	0.53
	Retinol (µg/day)								
	T1 (<182.5)	194	68	1.00		114	43	1.00	
	T2 (182.5–427.8)	208	65	0.70 (0.40–1.22)		124	48	1.11 (0.49–2.52)	
	T3 (≥427.8)	195	72	0.95 (0.42–2.14)	0.76	115	46	1.63 (0.84–3.16)	0.11
	Vitamin D (µg/day) ^b^								
	T1 (<1.9)	191	67	1.00		102	36	1.00	
	T2 (1.9–6.2)	190	65	0.67 (0.35–1.25)		114	53	1.84 (0.70–4.81)	
	T3 (≥6.2)	216	73	0.91 (0.56–1.48)	0.73	137	48	0.72 (0.35–1.44)	0.08
	β-Carotene (µg/day)								
	T1 (<554.8)	202	73	1.00		118	51	1.00	
	T2 (554.8–1836.6)	207	73	0.89 (0.44–1.81)		119	46	0.83 (0.48–1.44)	
	T3 (≥1836.6)	188	59	0.93 (0.52–1.66)	0.86	116	50	0.58 (0.30–1.13)	0.15
	Total carotenoids (µg/day)								
	T1 (<3464.6)	211	77	1.00		117	46	1.00	
	T2 (3464.6–10075.0))	196	62	0.83 (0.47–1.46)		119	46	0.89 (0.40–1.97)	
	T3 (≥10075.0)	190	66	1.04 (0.57–1.90)	0.77	117	45	0.76 (0.25–2.34)	0.65
White participants	α-Tocopherol (mg/day)								
	T1 (<6.1)	370	126	1.00		197	54	1.00	
	T2 (6.1–10.2)	274	102	1.17 (0.73–1.89)		202	54	0.80 (0.39–1.65)	
	T3 (≥10.2)	274	91	0.56 (0.25–1.27)	0.11	211	62	0.51 (0.24–1.10)	0.09
	Retinol (µg/day)								
	T1 (<246.2)	308	106	1.00		190	50	1.00	
	T2 (246.2–491.2)	266	101	0.83 (0.41–1.71)		207	54	0.64 (0.39–1.05)	
	T3 (≥491.2)	344	112	0.82 (0.37–1.81)	0.63	213	66	0.60 (0.25–1.46)	0.33
	Vitamin D (µg/day) ^b^								
	T1 (<2.9)	282	96	1.00		201	56	1.00	
	T2 (2.9–15.9)	284	109	1.24 (0.76–2.04)		227	60	0.70 (0.41–1.19)	
	T3 (≥15.9)	352	114	0.77 (0.40–1.48)	0.24	182	54	1.34 (0.63–2.85)	0.18
	β-Carotene (µg/day)								
	T1 (<411.1)	375	129	1.00		188	53	1.00	
	T2 (411.1–1600.6)	296	111	1.81 (1.04–3.17)		194	58	1.23 (0.67–2.24)	
	T3 (≥1600.6)	247	79	0.81 (0.56–1.16)	0.01	228	59	1.14 (0.63–2.05)	0.90
	Total carotenoids (µg/day)								
	T1 (<3005.4)	375	128	1.00		181	53	1.00	
	T2 (3005.4–9489.2)	294	103	0.91 (0.45–1.86)		204	52	1.15 (0.45–2.90)	
	T3 (≥9489.2)	249	88	0.96 (0.58–1.58)	0.77	225	65	1.50 (0.53–4.04)	0.65

^a^ Odds ratio (OR), 95% confidence interval (95%CI) and *P_trend_* values were estimated by logistic regression (Proc Survey Logistic). Analyses were adjusted for age, sex, education, body mass index, smoking status, total energy intake, alcohol drinking habits, hepatitis B virus infection, hepatitis C virus infection, history of diabetes and history of high blood pressure. For all participants, the model was additionally adjusted for race/ethnicity; ^b^ Daily vitamin D intake includes vitamin D intake from diet and supplement.

## Data Availability

Publicly available datasets were analyzed in this study. This data can be found here: https://wwwn.cdc.gov/nchs/nhanes/ (accessed on 6 July 2021).
